# Albumin inhibits the nuclear translocation of Smad3 via interleukin-1beta signaling in hepatic stellate cells

**DOI:** 10.1038/s41598-021-82758-4

**Published:** 2021-02-04

**Authors:** Ji Hoon Park, Janghyun Kim, So-Young Choi, Boram Lee, Jung-Eun Lee, Heekyung Park, Ji Wook Moon, Sun-Hwa Park, Jae Min Lee, Hong Sik Lee, Junseo Oh

**Affiliations:** 1 Protein Drug Team at New Drug Development Center, Osong Medical Innovation Foundation, Osong, 28160 Korea; 2 Department of Anatomy, Korea University College of Medicine, Seoul, 02841 Korea; 3 Department of Internal Medicine, Korea University College of Medicine, Seoul, 02841 Korea; 4 Department of Biomedical Science, Korea University Graduate School, Seoul, 02841 Korea

**Keywords:** Drug development, Molecular medicine, Cell signalling, Mechanisms of disease

## Abstract

Activation of quiescent hepatic stellate cells (HSCs) to myofibroblasts plays a key role in liver fibrosis. We had previously shown that albumin and its derivative, R-III (a retinol-binding protein—albumin domain III fusion protein), inhibited HSC activation by sequestering retinoic acid (RA) and that R-III administration reduced carbon tetrachloride (CCl_4_)-induced liver fibrosis. In this study, we aimed to elucidate the mechanism of action of albumin downstream of RA sequestration. Nuclear factor-κB p65 was evenly distributed in the cytoplasm in activated mouse HSCs, whereas albumin expression or R-III treatment (albumin/R-III) caused the nuclear translocation of p65, probably via RA sequestration, resulting in a dramatic increase in interleukin-1beta (IL-1β) expression. Albumin/R-III in turn induced the phosphorylation of Smad3 at the linker region, inhibiting its nuclear import in an IL-1β-dependent manner. Consistent with the in vitro results, the level of IL-1β mRNA expression was higher in CCl_4_/R-III-treated livers than in CCl_4_-treated livers. These findings reveal that albumin/R-III inhibits the transforming growth factor-β-Smad3 signaling as well as the retinoic acid receptor-mediated pathway, which probably contributes to the inhibition of HSC activation, and suggest that R-III may be an anti-fibrotic drug candidate.

## Introduction

Stellate cells (SCs), commonly known as hepatic stellate cells (HSCs), were first mentioned by Von Kupffer in 1876^1^. HSCs are pericytes found in the perisinusoidal space of the liver and constitute approximately 5–8% of total liver cells^2^. They are present in a quiescent state, exhibit non-proliferative characteristics in the normal liver, and store approximately 80% of the total body content of vitamin A (retinol) as retinyl esters in cytoplasmic lipid droplets. In response to fibrogenic stimuli, the quiescent SCs undergo functional and phenotypic changes, referred to as “activation,” and transform into myofibroblast-like cells^3^. This process is characterized by a loss of the vitamin A-containing cytoplasmic lipid droplets, high cellular proliferation, positive staining for alpha-smooth muscle actin (α-SMA), and an enhanced synthesis of extracellular matrix proteins. When cultured on plastic, HSCs undergo spontaneous activation in vitro. It is widely accepted that the activation of HSCs plays a critical role in liver fibrosis, which is characterized by an excessive deposition of extracellular matrix components^4^. Cells resembling HSCs were isolated from the pancreas in the late 1990s^5^, and in a manner similar to HSCs, these pancreatic SCs were also found to play an important role in pancreatic fibrogenesis^6^. Thus, SCs are considered an attractive target for anti-fibrotic therapies^7^. However, despite extensive investigations, there is as yet no effective therapy for liver fibrosis and end-stage cirrhosis, except for the removal of the causative agent and organ transplantation^8^.

Retinoids (vitamin A and its metabolites) regulate multiple physiological activities, such as vision, cell proliferation and differentiation^9^. Studies have shown that retinoids exert most of their effects primarily through their binding to nuclear receptors, retinoic acid receptors (RARs) and retinoid X receptors^10^. Vitamin A, acquired from the diet, is transported to the liver and taken up by hepatocytes as a chylomicron remnant. It has been suggested that retinol-binding protein (RBP) plays a role in the transfer of retinol from hepatocytes to HSCs via an RBP receptor, STRA6^11^. It is likely that upon HSC activation, the cytoplasmic lipid droplets collapse and a portion of the retinoid contents is released and metabolized into retinaldehyde, which is further irreversibly oxidized to retinoic acid (RA) by retinaldehyde dehydrogenase. This is supported by the fact that the RA level is increased in activated SCs compared with that in pre-activated SCs, whereas the retinyl ester and retinol contents are decreased^12,13^. To examine the role of RA, researchers have assessed the effects of exogenous RA on HSCs and liver fibrosis, but the results have been controversial, with several studies showing that RA inactivated HSCs and alleviated hepatic fibrosis^14,15^ and other reports showing the opposite effects^16,17^. Our recent study suggested that endogenous RA plays a role in HSC activation^18^.

Albumin, an abundant multifunctional plasma protein, is synthesized primarily by liver cells^19^. It is composed of three homologous domains (I, II, and III), each of which is formed by two subdomains (A and B), and binds a wide variety of hydrophobic ligands, including fatty acids and retinoids^20,21^. In a previous study, we showed that albumin was endogenously expressed in quiescent SCs, but not in activated SCs, and that its forced expression in the activated SCs induced the phenotypic conversion of myofibroblasts into fat-storing, early-activated cell phenotype^22^. On the basis of this finding, we developed a recombinant fusion protein (designated R-III) as an anti-fibrotic agent, in which the domain III of albumin was fused to the C-terminus of RBP^23^. We had selected RBP for targeted delivery to SCs as RBP and its membrane receptor (STRA6) coordinate the cellular uptake of retinol into HSCs^11^. Our follow-up study showed that R-III inhibited HSC activation in vitro and reduced liver and kidney fibrosis in vivo^18,24^. In this present study, we aimed to examine the modes of action of albumin and R-III. We found that RA sequestration by albumin expression or R-III treatment led to the downregulation of transforming growth factor-β (TGF-β)-Smad3 signaling in HSCs.

## Results

### Involvement of retinoic acid in the process of HSC activation

**Figure 1:**
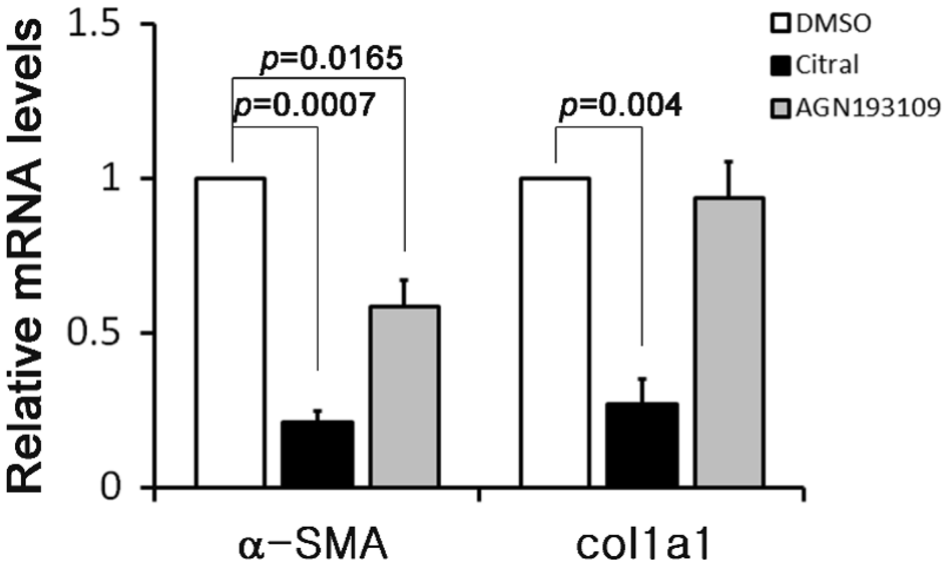
Retinoic acid plays a role in hepatic stellate cell activation. Hepatic stellate cells after passage 1 were treated with citral (100 μM) or AGN193109 (1 μM) for 20 h, and the alpha-smooth muscle actin (α-SMA) and collagen type I alpha 1 (col1a1) expression levels were then analyzed by real-time PCR. The data represent the means ± SD for three independent experiments. *P*-value, paired *t*-test (compared with untreated HSCs-P1).

We had previously shown that albumin expression or R-III treatment (albumin/R-III) inactivated HSCs by sequestering RA and that, as assessed by western blot analysis for α-SMA, a well-known marker for HSC activation, RAR-mediated signaling might be involved in HSC activation^18^. To examine the role of RA more closely, HSCs after passage 1 (HSCs-P1; activated HSCs) were treated with either citral (a retinaldehyde dehydrogenase inhibitor that blocks RA biosynthesis) or AGN193109 (an RAR antagonist), and the mRNA expression levels of α-SMA and collagen type I (both reliable markers of activated HSCs) in the cells were then analyzed by real-time PCR. Citral significantly reduced the mRNA levels of both α-SMA (Acta2) and collagen type I alpha 1 (Col1a1), whereas AGN193109 affected the expression of α-SMA only (Fig. 1). As previously reported^18^, citral treatment induced phenotypic conversion from activated/myofibroblastic phenotype to a fat-storing phenotype and did not appear to be toxic. This finding suggests that RA, required for HSC activation, exerts its effects mainly through as-yet unidentified pathways rather than via its nuclear receptor, RAR.

### Effect of albumin/R-III on the nuclear translocation of nuclear factor-κB (NF-κB) in HSCs

**Figure 2:**
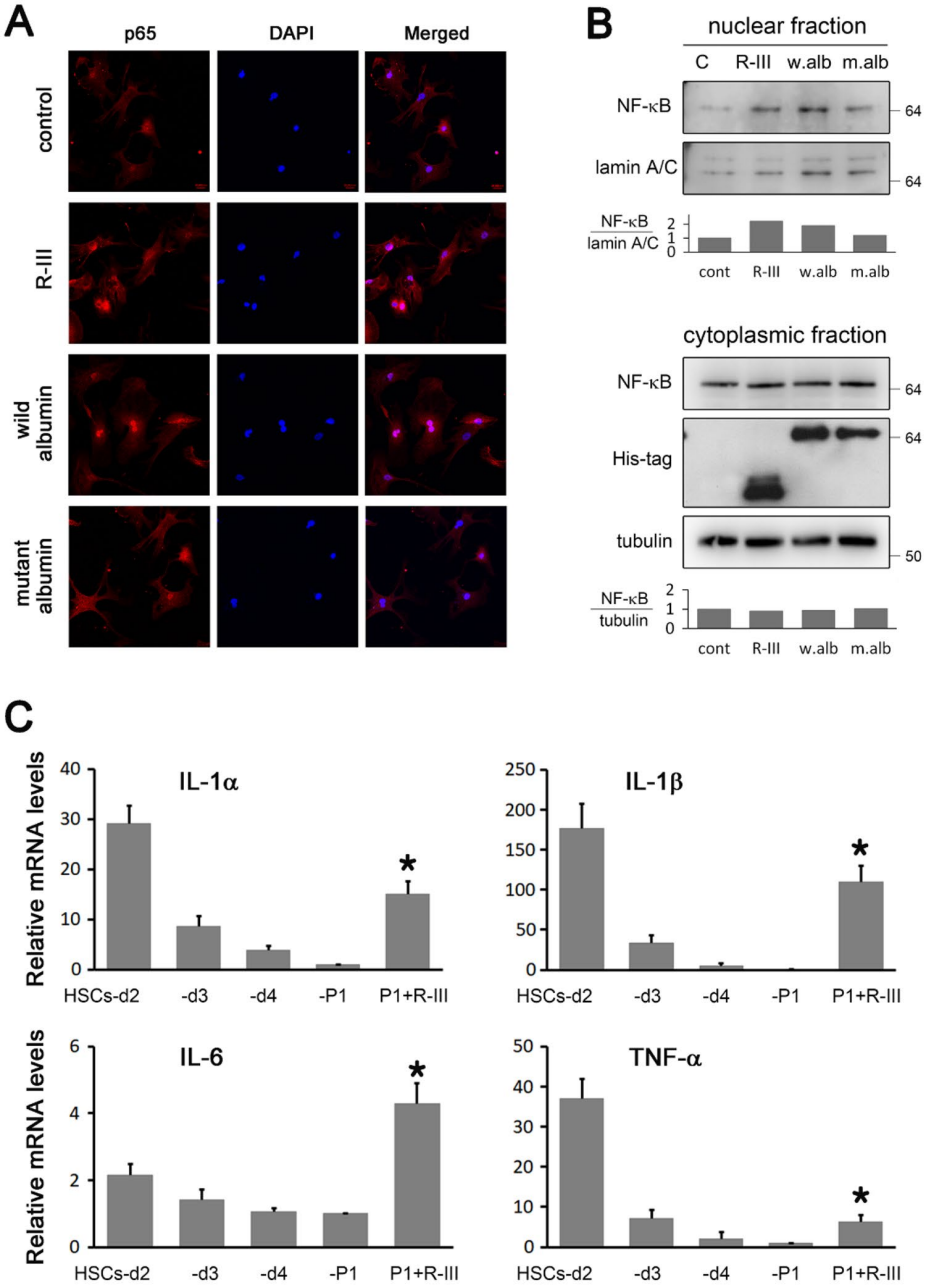
R-III treatment and albumin expression promote the nuclear translocation of NF-κB. (**A**, **B**) Hepatic stellate cells after passage 1 (HSCs-P1) were treated with HPLC-purified, His-tagged R-III (0.25 μM) or transfected with plasmids encoding His-tagged, wild-type or mutant albumin (R410A, Y411A, K525A) and then analyzed by immunofluorescence using the anti-p65 antibody (**A**). The cell nuclei were stained with DAPI (blue). (**B**) Nuclear and cytoplasmic fractions were prepared from the treated cells and subjected to western blotting using anti-NF-κB antibody. Lamin A/C and α-tubulin were used as internal controls. Bar graph represents the average of two independent experiments. Full-length blots are presented in Supplementary Fig. S4. (**C**) Total RNA was isolated from the HSCs on days 2, 3, and 4 after seeding (different stages of activation), and HSCs-P1 with or without R-III treatment, and the expression levels of IL-1α, IL-1β, IL-6, and TNF-α were then analyzed by real-time PCR. *P < 0.05, paired *t*-test (*n* = 3) (compared with untreated HSCs-P1).

As previous studies have shown that RA exerts an anti-inflammatory effect by inhibiting the nuclear translocation of NF-κB in microglial and neuronal cells^25,26^, we examined the subcellular localization of NF-κB in HSCs. Immunofluorescence analysis revealed that NF-κB p65 was evenly distributed in the cytoplasm in HSCs-P1, whereas its nuclear translocation was promoted by albumin/R-III (Fig. 2A). By contrast, the nuclear translocation of NF-κB p65 was not detected in HSCs-P1 expressing the mutant albumin (R410A, Y411A, K525A), in which three high-affinity fatty acid-binding sites were substituted for alanine and whose expression failed to sequester RA^18^. Western blot analysis of the nuclear protein extracts also showed similar trend (Fig. 2B). Taken together with previous findings that the RA level is increased during HSC activation but decreased after albumin/R-III treatment^13,18^, this suggests that RA also blocks the nuclear import of NF-κB in HSCs. Indeed, real-time PCR showed that the expression levels of the inflammation-related genes *IL-1α*, *IL-1β*, *IL-6*, and tumor necrosis factor-alpha (*TNF-*α) were decreased during the culture activation of HSCs (HSCs on days 2, 3, and 4 after seeding, and HSCs-P1) but increased after R-III treatment in HSCs-P1 (Fig. 2C).

### IL-1β mediation of the R-III effect on HSC activation

**Figure 3:**
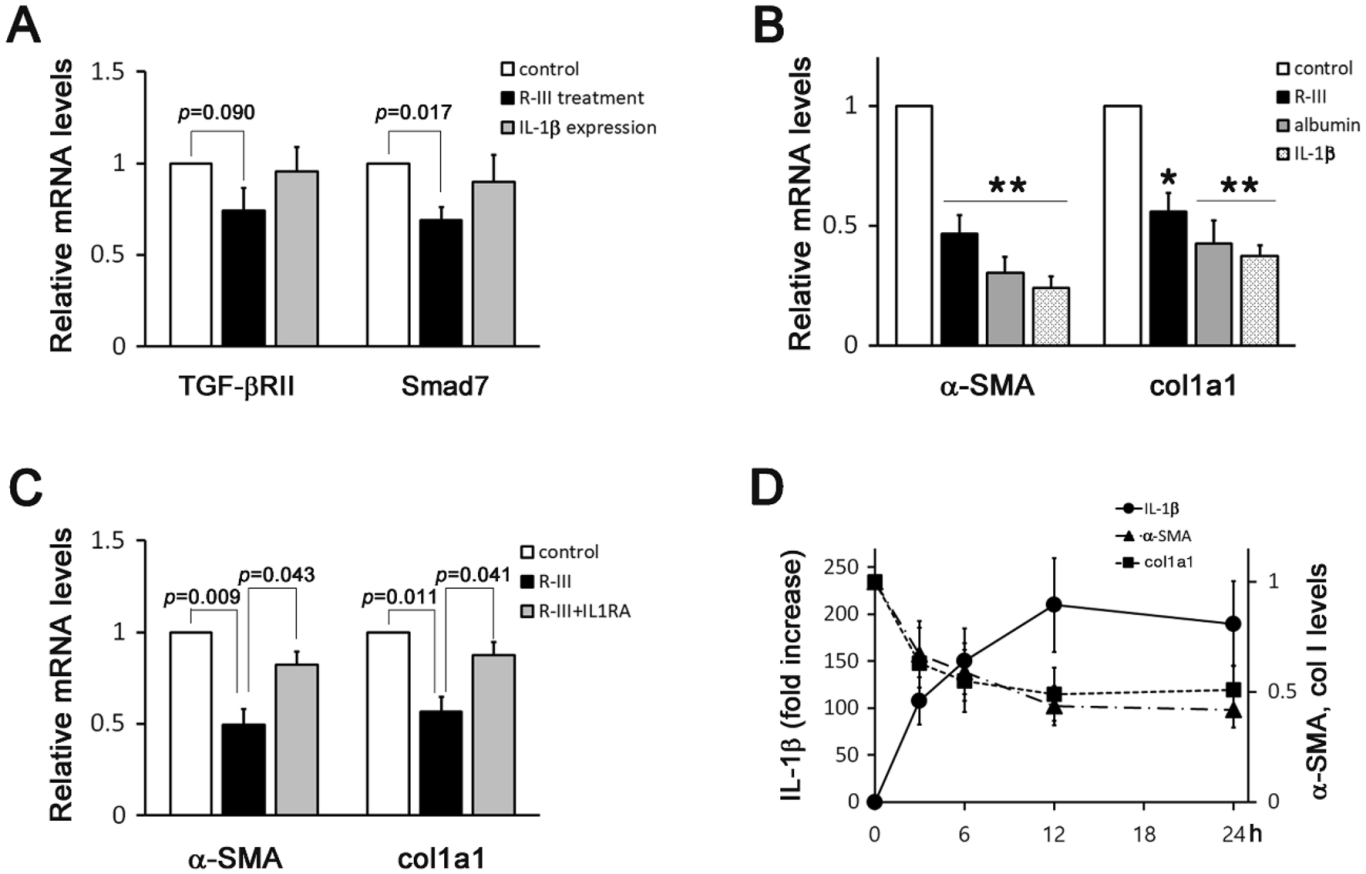
Interleukin (IL)-1β mediates the inhibitory effect of R-III on hepatic stellate cell activation. (**A**) Hepatic stellate cells after passage 1 (HSCs-P1) were treated with R-III (0.25 μM) for 20 h or transfected with a plasmid encoding IL-1β, and the TGF-β receptor type II (TGF-βRII) and Smad7 expression levels were then analyzed by real-time PCR. (**B**) HSCs-P1 were treated with R-III for 20 h or transfected with a plasmid encoding albumin or IL-1β, and the α-SMA and collagen type I alpha 1 (col1a1) expression levels were then analyzed by real-time PCR. (**C**) HSCs-P1 were treated with R-III in the presence or absence of IL-1RA (1 μg/mL), and the α-SMA and col1a1 expression levels were then analyzed by real-time PCR. (**D**) HSCs-P1 were treated with R-III and harvested at the indicated time points, and the IL-1β, α-SMA, and col1a1 expression levels were then analyzed by real-time PCR. Left y-axis: fold changes of IL-1β mRNA; right y-axis: α-SMA and col1a1 mRNA levels. *P < 0.05, **P < 0.01, paired *t*-test (*n* = 3) (compared with untreated HSCs-P1).

Although several studies have suggested that the inflammatory mediators play pro-fibrogenic roles in liver fibrosis^27^, it was also reported that IL-1β neutralizes the activity of TGF-β in different cells through different mechanisms, such as TGF-β receptor type II (TGF-βRII) downregulation, Smad7 upregulation, and Smad3 inhibition^28–30^. First, we examined whether IL-1β expression or R-III treatment (via inducing IL-1β) affects the expression of TGF-βRII and Smad7 in HSCs-P1. Real-time PCR revealed that only Smad7 was slightly decreased by R-III and that IL-1β expression did not affect TGF-βRII and Smad7 expression (Fig. 3A, Supplementary Fig. S1). We then tested the possibility that IL-1β expression may inhibit HSC activation, a process in which TGF-β signaling plays an important role. Surprisingly, IL-1β expression in HSCs-P1 downregulated the mRNA expression of α-SMA and collagen type I, as was previously seen with albumin/R-III^22^ (Fig. 3B). In addition, when HSCs-P1 were treated with R-III in the presence of IL-1 receptor antagonist (IL-1RA), the R-III effect was significantly reduced, suggesting that IL-1β mediates, at least in part, the R-III effect on HSC activation (Fig. 3C). The time course of changes in the mRNA levels of IL-1β, α-SMA, and collagen type I was examined after R-III treatment of the HSCs-P1. R-III induced a steady increase in the IL-1β mRNA level with a peak in expression at ~ 12 h (> 100 fold), and simultaneously downregulated the expression of both α-SMA and collagen type I (Fig. 3D). Thus, these findings suggest that RA sequestration by albumin/R-III promotes the nuclear translocation of NF-κB and that the resulting IL-1β may mediate the inhibitory effect of R-III on HSC activation.

### Effect of albumin/R-III on the nuclear translocation of Smad3

**Figure 4:**
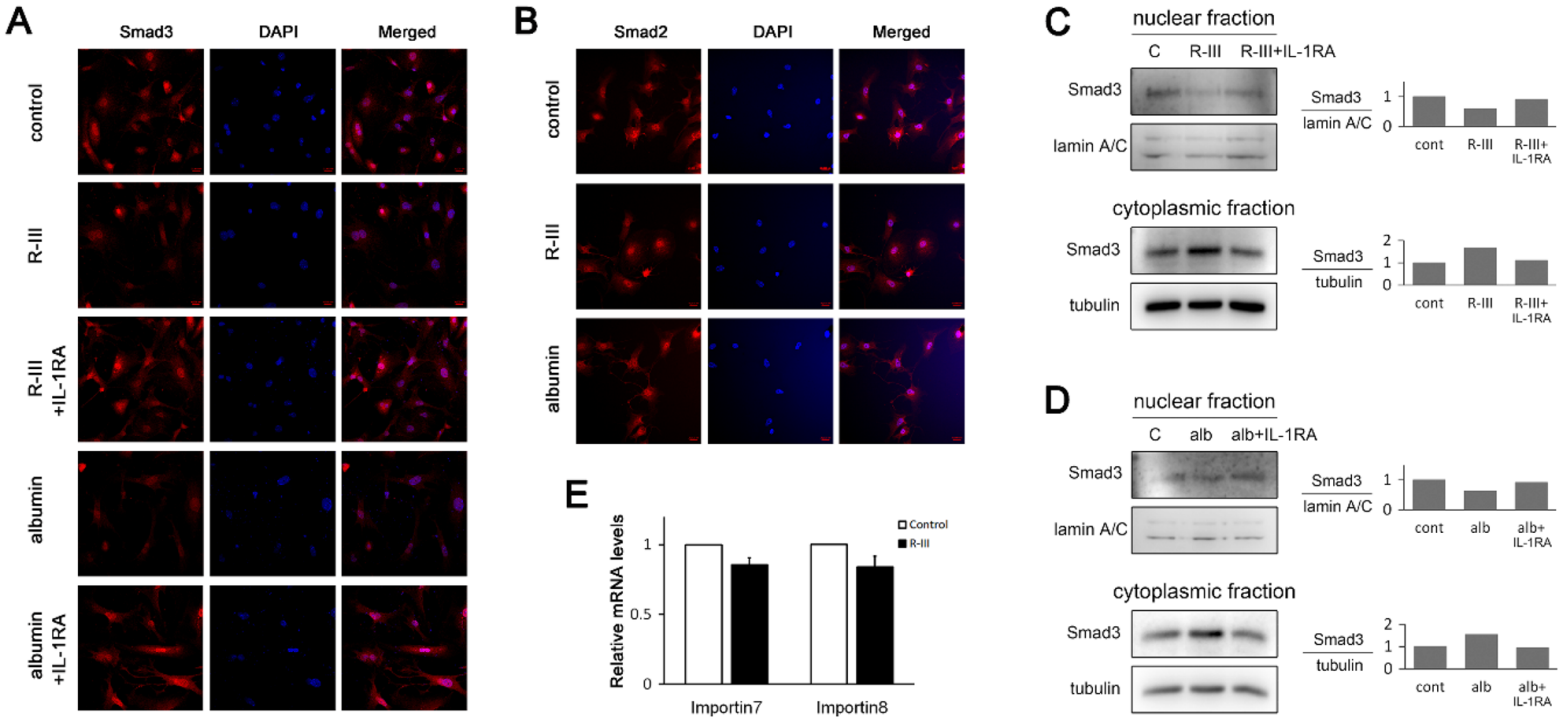
R-III treatment and albumin expression inhibit the nuclear translocation of Smad3 in hepatic stellate cells. (**A**–**D**) Hepatic stellate cells after passage 1 (HSCs-P1) were treated with R-III (0.25 μM) or transfected with an albumin expression vector in the presence or absence of interleukin (IL)-1RA and then analyzed by immunofluorescence using antibodies against Smad3 (**A**) or Smad2 (**B**). Nuclear and cytoplasmic fractions were prepared from the R-III-treated (**C**) and albumin-transfected cells (**D**) and subjected to western blotting using anti-Smad3 antibody. Bar graph represents the average of two independent experiments. Full-length blots are presented in Supplementary Fig. S4. (**E**) HSCs-P1 were treated with R-III, and the importin 7 and importin 8 expression levels were then analyzed by real-time PCR.

IL-1β reportedly inhibited the nuclear translocation of Smad proteins through the phosphorylation of the Smad linker region in mesenchymal stem cells^30^. Therefore, we tested whether albumin/R-III affects the subcellular distribution of Smads. Immunofluorescence analysis revealed that both Smad2 and Smad3 were accumulated in the nucleus in HSCs-P1, whereas the nuclear import of Smad3, but not of Smad2, was inhibited by albumin/R-III in an IL-1β-dependent manner (Fig. 4A,B). Western blot analysis of the nuclear protein extracts also showed inhibitory effects on Smad3 nuclear import (Fig. 4C,D). We then examined the expression of importin 7 and importin 8, which are responsible for transporting phosphorylated Smad2 and Smad3 into the nucleus^31^, but found no significant changes in their levels with R-III treatment (Fig. 4E).

### Albumin/R-III promotion of Smad3 linker phosphorylation via IL-1β

**Figure 5:**
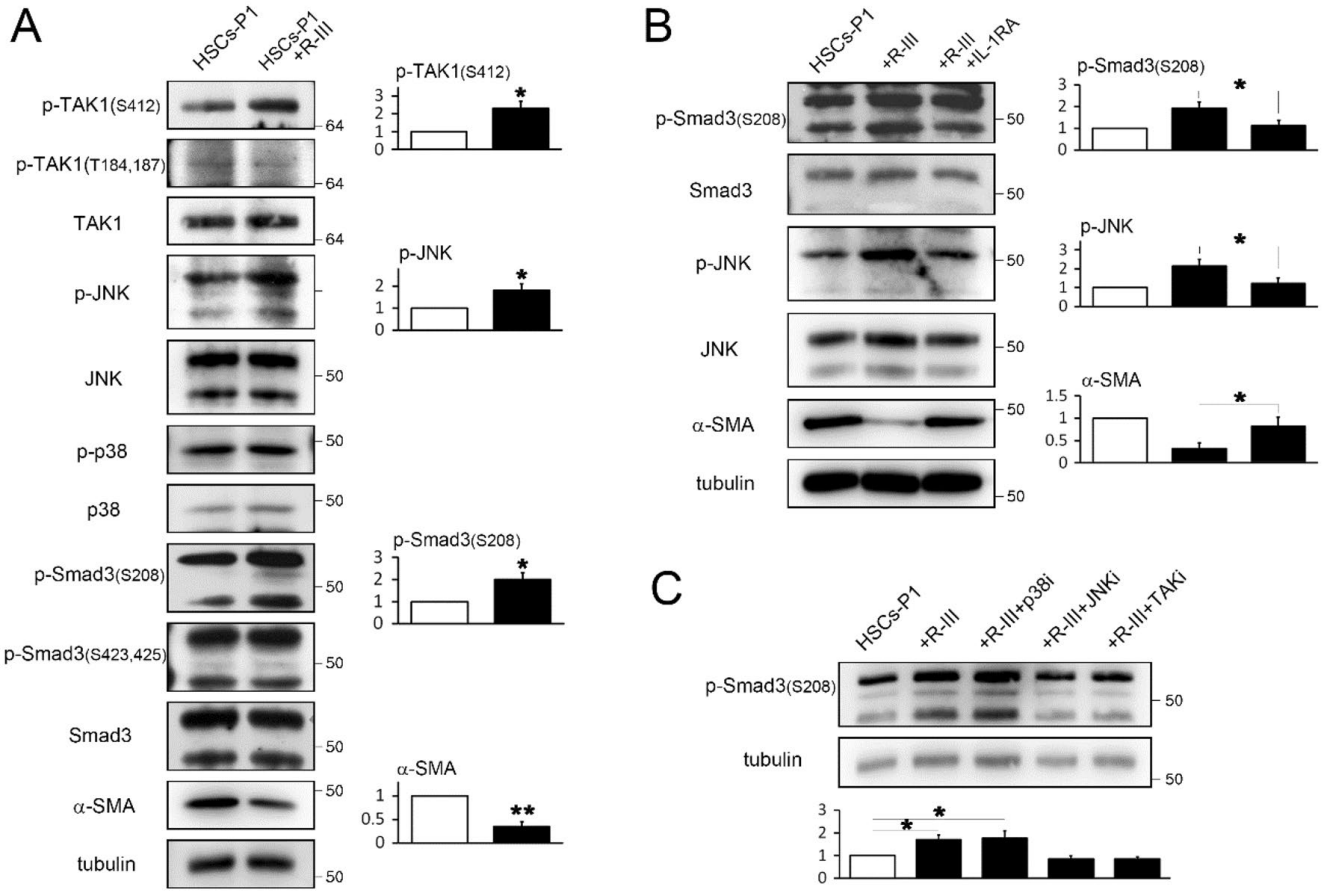
R-III treatment induces the phosphorylation of the Smad3 linker region IL-1β-dependently. (**A**) Hepatic stellate cells after passage 1 (HSCs-P1) were treated with R-III (0.25 μM) for 20 h, and the cell lysates were then analyzed by western blotting. The quantitative densitometric data are presented as mean fold change ± SD of three independent experiments. *P*-value, paired *t*-test (compared with untreated cells). *P < 0.05, **P < 0.01 (**B**) HSCs-P1 were treated with R-III in the presence or absence of interleukin (IL)-1RA (1 μg/mL), and the cell lysates were then analyzed by western blotting. (**C**) HSCs-P1 were treated with R-III in the presence of an inhibitor for TAK1 ((5Z)-7-oxozeaenol, 0.25 μM), p38 (SB203580, 5 μM), or JNK (SP600125, 10 μM), and the cell lysates were then analyzed by western blotting. α-tubulin was used as a loading control. Full-length blots are presented in Supplementary Fig. S4.

**Figure 6:**
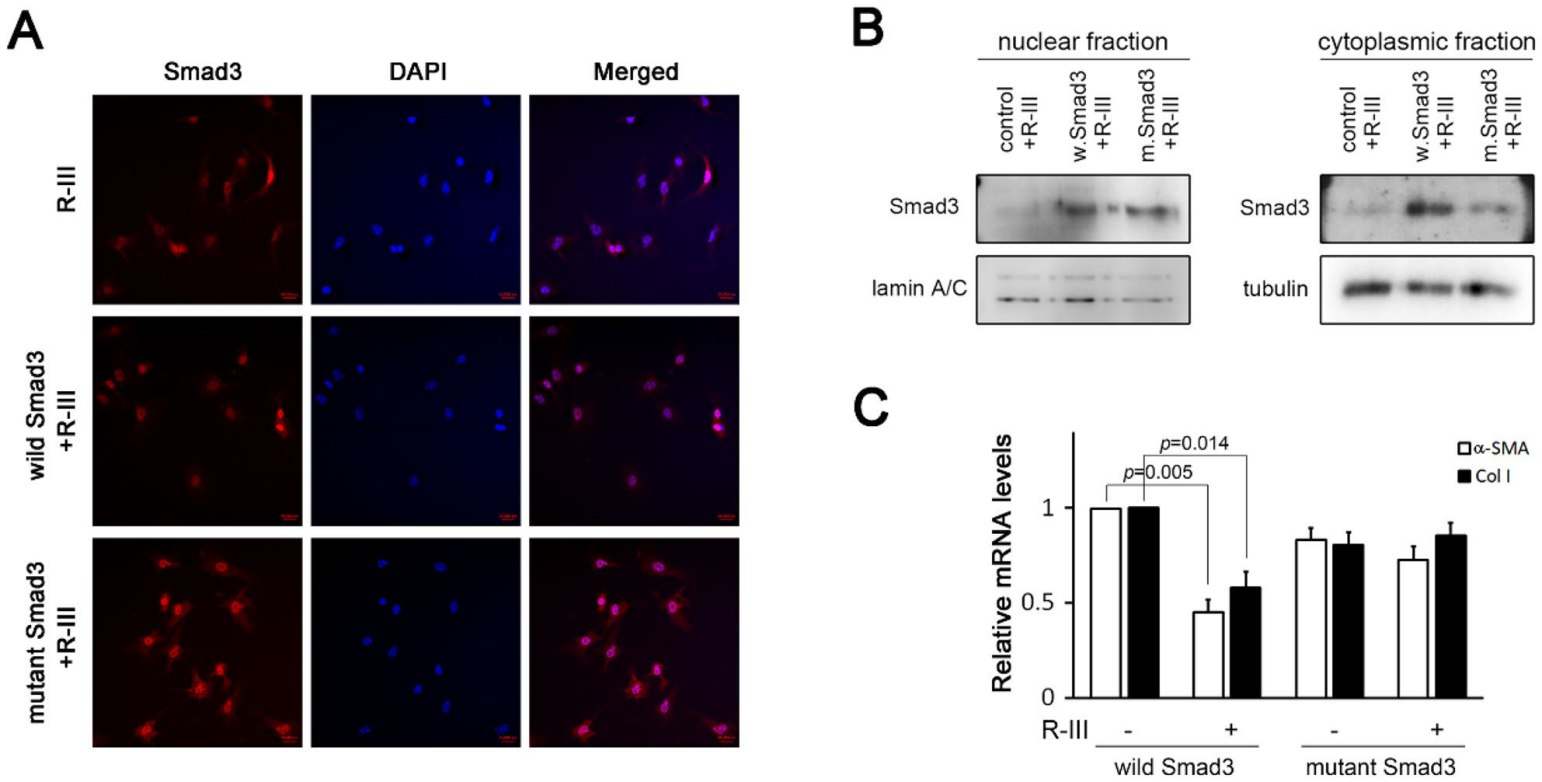
Smad3 linker phosphorylation affects its nuclear translocation in hepatic stellate cells. (**A**–**C**) Hepatic stellate cells after passage 1 (HSCs-P1) were transfected with empty vector or with an expression plasmid encoding wild-type or mutant Smad3 (S204A, S208A, S213A) and then treated with R-III (0.25 μM) for 20 h. (**A**) The treated cells were analyzed by immunofluorescence using anti-Smad3 antibody. (**B**) Nuclear and cytoplasmic fractions were prepared from the treated cells and subjected to western blotting using anti-Smad3 antibody. Full-length blots are presented in Supplementary Fig. S4. (**C**) HSCs-P1 were transfected with an expression plasmid encoding wild-type or mutant Smad3 and then treated with or without R-III. The α-SMA and collagen type I (col I) expression levels in the treated cells were analyzed by real-time PCR. *P*-value, paired *t*-test (*n* = 3) (compared with untreated HSCs-P1).

TGF-β-activated kinase 1 (TAK1) and its downstream targets, such as p38 and JNK, have been shown to phosphorylate Smad3 in the linker region and inhibit its nuclear translocation^32,33^. Therefore, we carried out several experiments to elucidate the mechanism through which albumin/R-III inhibits the nuclear import of Smad3 IL-1β-dependently. Western blot analysis revealed that albumin/R-III in HSCs-P1 led to an increased phosphorylation of TAK at Ser412, which is required for TAK1-mediated IL-1R signaling^34^, whereas phosphorylation at the TAK1 autophosphorylation sites T184 and T187 was not significantly changed (Fig. 5A, Supplementary Fig. S2). Albumin/R-III also enhanced the level of p-JNK, but not that of p-p38, and increased the phosphorylation of Smad3 linker region at S208. However, the C-terminal phosphorylation of Smad3 (S423, S425) was not affected. Importantly, these R-III effects on Smad3, JNK, and α-SMA were abrogated by IL-1RA (Fig. 5B), suggesting that albumin/R-III-mediated IL-1β signaling induces the phosphorylation of the Smad3 linker region. When HSCs-P1 were treated with R-III in the presence of the inhibitor for p38 (SB203580), JNK (SP600125), or TAK1 ((5Z)-7-oxozeaenol), the phosphorylation of the Smad3 linker region was reduced by (5Z)-7-oxozeaenol and SP600125 (Fig. 5C). Lastly, to confirm the functional significance of Smad3 linker phosphorylation, we transfected HSCs-P1 with the expression vectors for wild-type or mutant Smad3 (S204A, S208A, S213A; in which three linker phosphorylation sites were substituted for alanine). The effects of R-III on the subcellular localization of Smad3 and the expression of α-SMA and collagen type I were not observed in the mutant Smad3-expressing HSCs (Fig. 6A–C). These findings suggest that the albumin/R-III-induced phosphorylation of the Smad3 linker region may affect the subcellular localization of the protein and consequently inhibit TGF-β-Smad3 signaling.

### Importance of RA sequestration and IL-1β signaling for albumin/R-III activity

**Figure 7:**
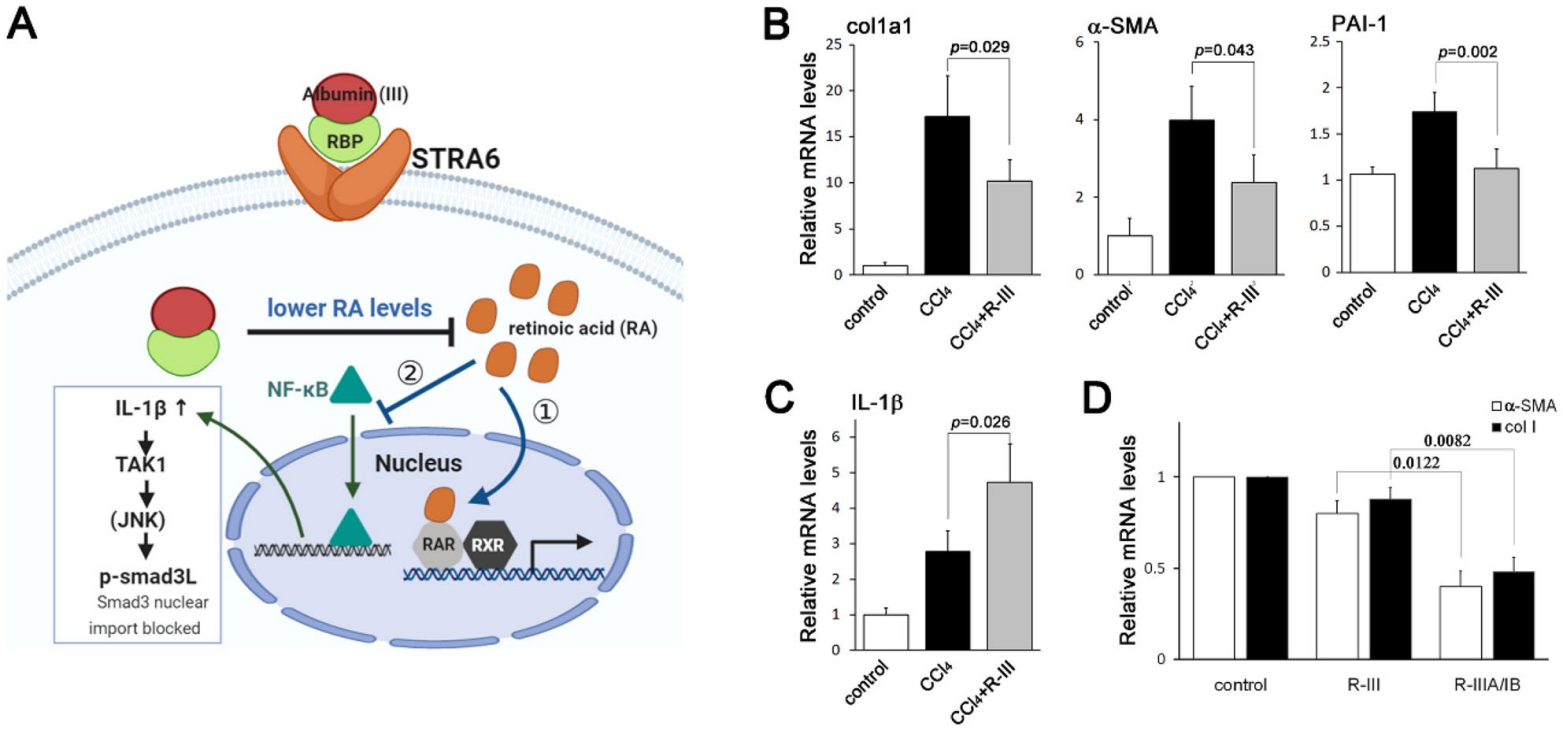
Retinoic acid sequestration is important for the anti-fibrotic activity of R-III. (**A**) Schematic drawing of the mechanism of action of R-III in hepatic stellate cells (HSCs). R-III enters HSCs via a retinol-binding protein (RBP) receptor STRA6 and sequesters retinoic acid (RA), which not only ① inhibits the retinoic acid receptor (RAR)-mediated pathway but also ② blocks the nuclear translocation of Smad3 via IL-1β signaling. (**B**, **C**) Total RNA was extracted from the livers of control mice, CCl_4_-treated mice (CCl_4_ administered three times per week over a period of 7 weeks), and CCl_4_/R-III-treated mice (R-III injected daily during the last 2 weeks of CCl_4_ treatment), and the expression levels of the collagen type I alpha 1 (col1a1), α-SMA, PAI-1 (**B**), and IL-1β (**C**) were analyzed by real-time PCR. *P*-value, two-sample *t*-test (*n* = 10). (**D**) Hepatic stellate cells after passage 1 were treated with R-III (0.1 μM) or R-IIIA/IB (0.1 μM) for 20 h, and the α-SMA and collagen type I expression levels were then analyzed by real-time PCR. **P < 0.01, paired *t*-test (*n* = 3) (compared with untreated HSCs-P1).

Our results suggest that RA sequestration by albumin/R-III downregulates not only the RAR-mediated pathway but also TGF-β-Smad3 signaling through the activity of IL-1β, which contributes to the inhibition of HSC activation (Fig. 7A). However, previous studies have shown that the IL-1β level is increased in the fibrotic liver, suggesting that this cytokine may play a pro-fibrogenic role^27,35^. To verify our in vitro findings, we extracted RNA from the livers of (1) untreated (control) mice, (2) mice treated with carbon tetrachloride (CCl_4_) for 7 weeks (to induce liver fibrosis), and (3) mice treated with CCl_4_ for 7 weeks and administered with R-III during the last 2 weeks of CCl_4_ treatment for analysis by real-time PCR. Consistent with the findings of our previous study^18^, the mRNA level of collagen type I was increased in the fibrotic livers compared with that in the control livers but was reduced by R-III administration (Fig. 7B). Other Smad3 target genes, α-SMA and plasminogen activator inhibitor type-1 (PAI-1), also showed a similar pattern in mRNA expression. Importantly, the increased level of IL-1β mRNA in the CCl_4_-treated livers was further enhanced in the CCl_4_/R-III-treated livers (Fig. 7C). This finding corresponds to our in vitro results and supports the role of IL-1β in the anti-fibrotic effect of R-III in HSCs. Lastly, to reinforce the participation of RA sequestration in the actions of R-III, we developed a R-III derivative R-IIIA/IB, in which the IIIB subdomain of albumin was replaced by IB (Supplementary Fig. S3). As biophysical studies showed that retinoids form stable interactions with albumin domains IB and IIIA^21,36^, it is reasonable to speculate that R-IIIA/IB would sequester RA more efficiently. When HSCs-P1 were treated with a reduced concentration of R-III or R-IIIA/IB (0.1 μM), R-IIIA/IB reduced the mRNA levels of α-SMA and collagen type I more effectively (Fig. 7D). Therefore, our findings suggest that the IL-1β signaling resulting from RA sequestration plays a role in the inhibitory effect of R-III on HSC activation.

## Discussion

HSCs are considered an attractive target for anti-fibrotic therapies. Several previous studies have been carried out with the aim to inactivate HSCs and reduce liver fibrosis, but no effective therapy for treating liver fibrosis is currently available^8^. Furthermore, the molecular mechanism behind the activation of HSCs remains elusive, despite intensive research efforts over the last 30 years to elucidate this.

Although the role of RA in HSC activation has been proposed, previous reports about the effects of exogenous retinoids on HSCs and liver fibrosis were controversial^37^. Our recent study suggested that endogenous RA plays a role in HSC activation^18^. Herein, we have further demonstrated that RA is required for the maintenance of high levels of α-SMA and collagen type I expression in activated HSCs. Albumin/R-III caused the nuclear translocation of NF-κB, probably via RA sequestration, and the resulting IL-1β signaling in turn inhibited the nuclear translocation of Smad3, thereby downregulating the expression of Smad3 target genes, including those coding for α-SMA, collagen type I, and PAI-1. This explains why the mutant albumin (R410A, Y411A, K525A), whose expression failed to sequester RA, neither induced the nuclear translocation of NF-κB nor inhibited HSC activation^18,22^.

Our study suggests that endogenously expressed albumin acts to maintain the quiescent phenotype of pre-activated HSCs by sequestering RA. Then, it is plausible to assume that the mutations in the albumin gene, particularly those at the sites where RA binding occurs, would impair the ability of the albumin protein to sequester RA and consequently allow HSC activation. Such mutations may increase a person’s risk of developing liver fibrosis. It is notable that, according to the publicly available data at the cBioPortal for Cancer Genomics (https://www.cbioportal.org), K161, reported as an RA-binding site^21^, is the most frequently mutated amino acid among the albumin gene mutations found in hepatocellular carcinoma (HCC). Further study is required to address how mutation at K161 is linked to HCC, considering that cirrhosis, end stage of liver fibrosis, is a major risk factor of HCC.

Previous studies have suggested that inflammatory mediators play pro-fibrogenic roles in liver fibrosis^27^. Herein, we showed the conflicting role of IL-1β in HSCs, in that the cytokine inhibited TGF-β-Smad3 signaling and HSC activation. A direct inhibitory interaction between IL-1β and TGF-β signaling is not unprecedented, as it has been previously reported in osteoarthritic cartilage metabolism and fibroblast-to-myofibroblast transition^28,38^. A recent study also showed that IL-1β reduced the activation of human HSCs in vitro by assessing α-SMA mRNA levels^39^. This anti-fibrotic activity of IL-1β was also supported by our finding that its mRNA level was further increased in CCl_4_/R-III-treated livers in vivo compared with that in CCl_4_-treated livers. Although IL-1β has anti-fibrotic properties in HSCs, the benefits and risks of its use in the treatment of fibrosis should be carefully considered since IL-1β affects several different cell types.

Retinoid-storing SCs also exist in extrahepatic organs, such as the pancreas, kidneys, spleen, intestine, and lungs^40^. The SCs of these organs show striking similarities in morphology and perivascular location, which suggests that their activation may contribute to the myofibroblasts seen in fibrotic extrahepatic tissues^6^. As intravenously injected R-III was also detected in extrahepatic organs, such as the lungs, pancreas, kidneys, and intestine^23^, we are currently investigating whether R-III reduces extrahepatic fibrosis. In conclusion, we have elucidated the mode of action of albumin/R-III in HSC activation in this study and suggest that R-III, designed for SC targeting, may be a novel anti-fibrotic drug candidate.

## Materials and methods

### Materials

Male BALB/c mice were purchased from Orient Bio, Inc. (Sungnam, Korea) and maintained under temperature-, humidity-, and light-controlled conditions. All animal experimental protocols were approved by the local institutional review board (Korea University, College of Medicine) and performed in accordance with the NIH Guide for the Care and Use of Laboratory Animals. The fusion proteins R-III and RBP-albumin domain IIIA/IB (R-IIIA/IB) (Supplementary Fig. S3) were synthesized using a previously described method^18^. Citral, interleukin (IL)-1 receptor antagonist (IL-1RA), (5Z)-7-oxozeaenol, SB203580, and SP600125 were purchased from Sigma-Aldrich (St. Louis, MO, USA), and AGN193109 was purchased from Santa Cruz Biotechnology (Dallas, TX, USA).

### Isolation of mouse hepatic stellate cells

HSCs were isolated from male BALB/c mice (14 weeks old) as described previously^41^ with some modifications. In brief, the livers were perfused in situ with phosphate-buffered saline (PBS) and then with Gey’s balanced salt solution (GBSS) supplemented with collagenase (0.5 mg/ml; Sigma-Aldrich) and pronase (1 mg/ml; Sigma-Aldrich). The perfused livers were dissected, and the attached gall bladders and connective tissues were removed. The liver cell suspensions were further digested in GBSS supplemented with collagenase (0.25 mg/ml), pronase (0.5 mg/ml), and DNase (0.07 mg/ml; MP Biomedicals, Santa Ana, CA, USA), for 12 min in a 37 °C water bath. The cells were then washed and centrifuged in a 13.4% Nycodenz gradient at 1400×*g* for 20 min without brake. The interface containing the enriched HSCs was collected and washed with GBSS. Then, the isolated HSCs were cultured in Dulbecco’s modified Eagle’s medium supplemented with 10% fetal bovine serum. The purity of the HSCs was assessed by microscopic observation. The HSCs were passaged before reaching 70% confluence in the primary culture and used as activated HSCs. The activation status of the HSCs was assessed on the basis of their increased expression of α-SMA and collagen type I as well as through their morphologic changes.

### Quantitative real-time PCR

Total RNA was prepared using TRIzol (Ambion, Austin, TX, USA), and was used to synthesize the cDNA. Real-time PCR was performed on an ABI QuantStudio 3 Real-Time PCR system. To control for variations in the reaction, the PCR products were normalized against the mRNA levels of glyceraldehyde 3-phosphate dehydrogenase (*GAPDH*). The primers used are listed in Supplementary Table S1.

### Cell fractionation and western blot analysis

Cell lysates were prepared for analyses by electrophoresis and immunoblotting as described previously^18^. The primary antibodies used were raised against α-SMA (#A2547; Sigma-Aldrich); lamin A/C (#SC-376248; Santa Cruz Biotechnology); Smad2, phosphorylated (p)-Smad2 (S465, S467), Smad3, p-Smad3 (S423, S425), TGF-β-activated kinase 1 (TAK1), p-TAK1 (S412), p38, p-p38, JNK, p-JNK, and α-tubulin (#5339, #3101, #9523, #9520, #4505, #9339, #9212, #9211, #9252, #9255, and #2125, respectively; Cell Signaling Technology, Beverly, MA, USA); and p-Smad3 (S208) and p-TAK1 (T184, T187) (#PA5-38521 and #MA5-15073, respectively; Thermo Fisher Scientific, Waltham, MA, USA). Nuclear extraction from cultured cells were performed using a nuclear extract kit according to the instructions of the manufacturer (Cell Biolabs, Inc., San Diego, CA).

### Immunofluorescence

HSCs were seeded onto glass coverslips coated with gelatin. The cells were fixed with 4% paraformaldehyde and permeabilized with PBS containing 0.1% Triton X-100. The samples were then incubated overnight at 4 °C with the diluted primary antibody in 1% BSA in PBST (PBS + 0.1% Tween 20), followed by the Alexa Fluor 594-conjugated secondary antibody. The primary antibodies used were raised against Nuclear factor-κB (NF-κB) p65, Smad2, and Smad3 (#8242, #5339, and #9523, respectively; Cell Signaling Technology). After staining with 4′,6-diamidino-2-phenylindole (DAPI), the HSCs were observed under a Leica TCS SP8 microscope.

### Transfection

Transient transfection was performed with Lipofectamine 2000 according to the manufacturer’s protocol (Invitrogen, Carlsbad, CA, USA). 60–70% confluent cells were transfected using a DNA (μg) to lipofectamine (μl) ratio of 1:2 and harvested within 24 h.

### Statistical analysis

The results are expressed as the mean ± standard deviation (SD). The paired *t*-test or two-sample *t*-test was performed where appropriate. A *P*-value of less than 0.05 was considered statistically significant.

## Supplementary information

Supplementary Information.
