# Supplementary material for: Albumin inhibits the nuclear translocation of Smad3 via interleukin-1beta signaling in hepatic stellate cells
Source: Sci Rep. 2021 Feb 4;11:3196. doi: 10.1038/s41598-021-82758-4 (PMC7862402; doi:10.1038/s41598-021-82758-4)
Supplement: Supplementary file 1 — Supplementary Information. [file 41598_2021_82758_MOESM1_ESM.pdf]

Albumin inhibits the nuclear translocation of Smad3 via interleukin-1beta signaling in hepatic stellate cells

Ji Hoon Park[1,†], Janghyun Kim[2,†], So-Young Choi[1], Boram Lee[2], Jung-Eun Lee[2], Heekyung Park[2], Ji Wook Moon[2], Sun-Hwa Park[2], Jae Min Lee[3], Hong Sik Lee[3], and Junseo Oh[2,*]

[1]Protein Drug Team at New Drug Development Center, Osong Medical Innovation Foundation, Osong 28160, Korea

[2]Department of Anatomy, Korea University College of Medicine, Seoul 02841, Korea

[3]Department of Internal Medicine, Korea University College of Medicine, Seoul 02841, Korea

†These authors contributed equally to this work.

Correspondence: Junseo Oh, Department of Biomedical Science, Korea University Graduate School, Seoul 02841, Korea. Phone: +82 2 2286 1389; Fax: +82 2 2286 1387; E-mail: ohjs@korea.ac.kr

Supplementary Fig. S1. Hepatic stellate cells after passage 1 were transfected with either empty vector or expression plasmid for interleukin-1β (IL-1β), and the IL-1β expression level was then analyzed by real-time PCR. The data represent the means ± SD for three independent experiments. *P*-value, paired *t*-test (compared with control cells).

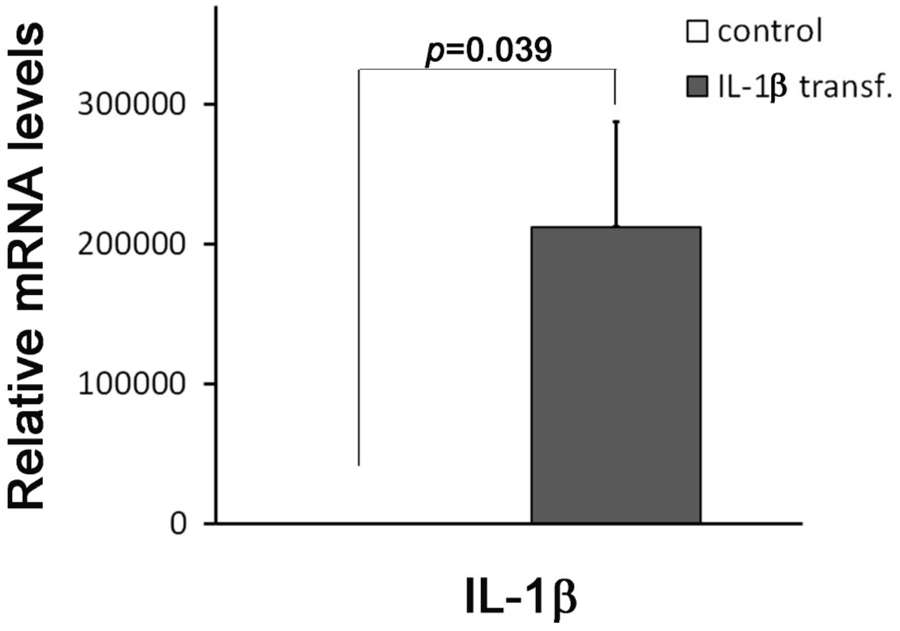

Supplementary Fig. S2. Albumin expression induces the phosphorylation of the Smad3 linker site. Hepatic stellate cells after passage 1 were transfected with the albumin expression vector, and the cell lysates were then analyzed by western blotting. Full-length blots are presented in Supplementary Fig. S4. The blots are representative of three independent experiments from separate cell preparations. α-tubulin was used as a loading control.

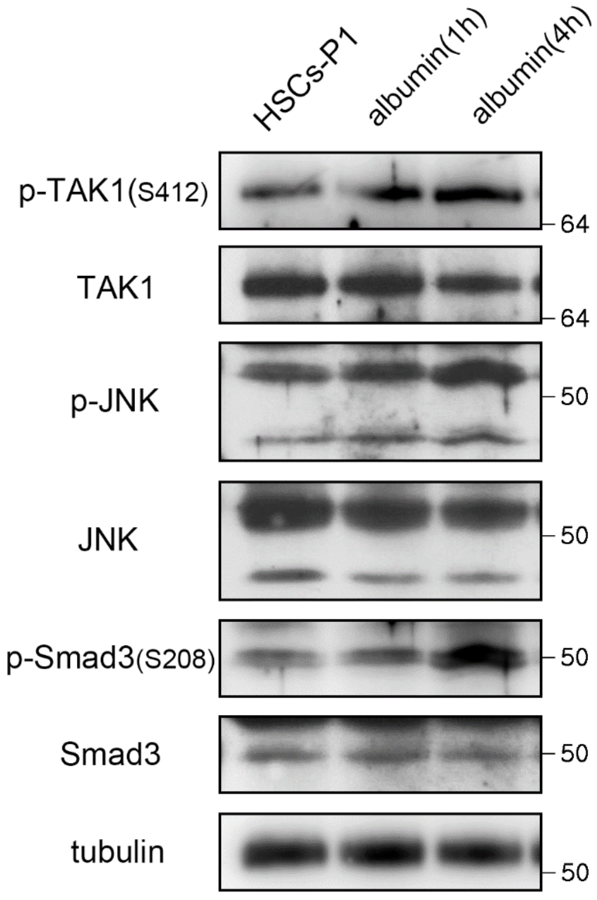

Supplementary Fig. S3. Schematic diagram of the retinol-binding protein (RBP)–albumin fusion proteins, R-III and R-IIIA/IB, in comparison with full-length albumin and RBP. The numbers indicate amino acids.

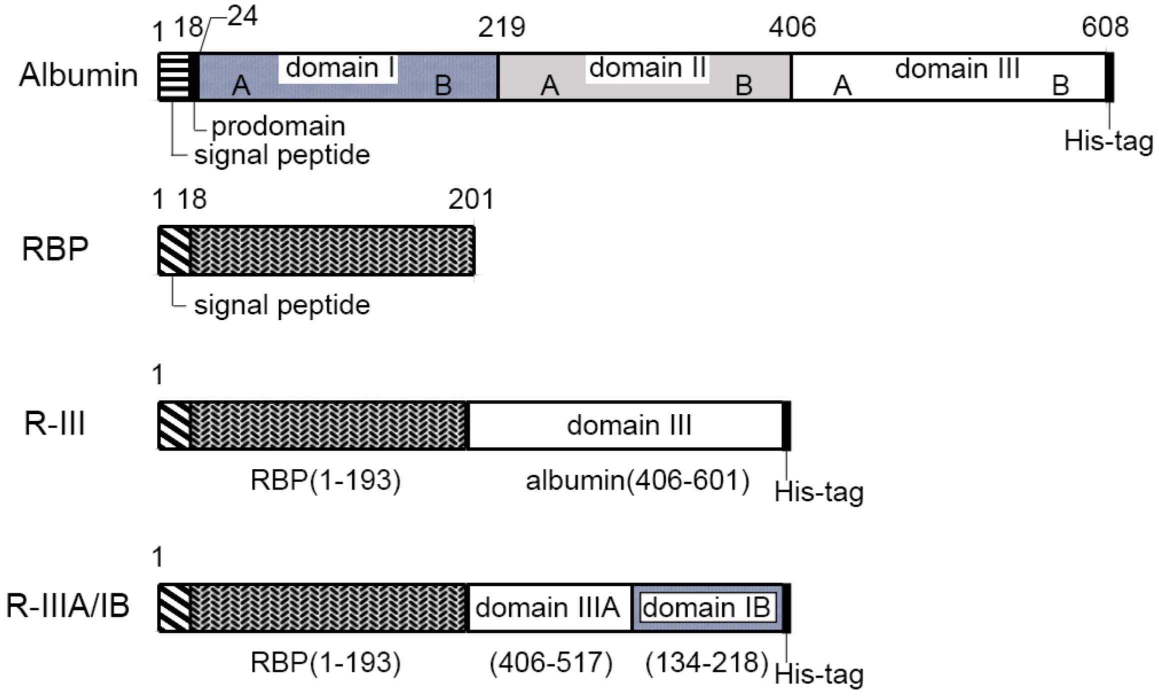

Supplementary Fig. S4. Uncropped full-length blot images.

Fig. 2B nuclear NF-kB

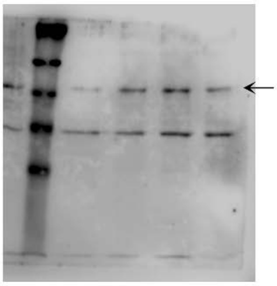

Fig. 2B nuclear lamin A/C

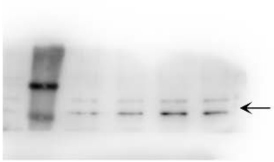

Fig. 2B cytoplasmic NF-kB

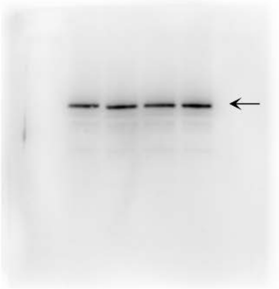

Fig. 2B cytoplasmic tubulin

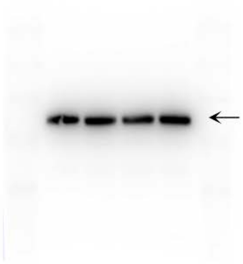

Fig. 4C nuclear Smad3

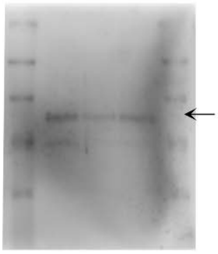

Fig. 4C nuclear lamin A/C

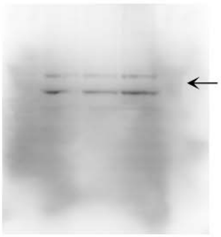

Fig. 4C cytoplasmic Smad3

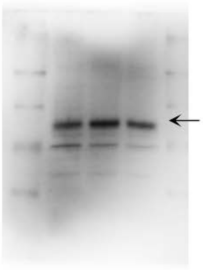

Fig. 4C cytoplasmic tubulin

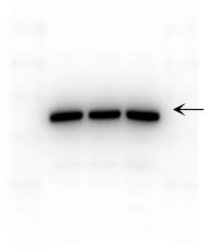

Fig. 4D nuclear Smad3

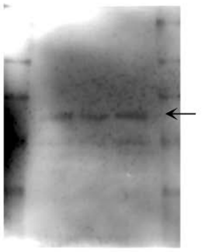

Fig. 4D nuclear lamin A/C

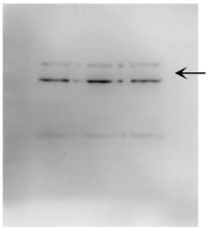

Fig. 4D cytoplasmic Smad3

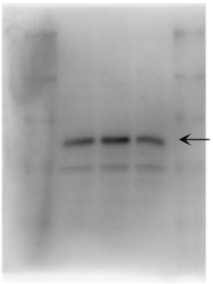

Fig. 4D cytoplasmic tubulin

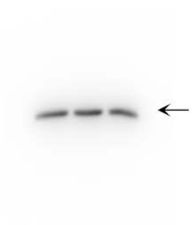

Fig. 5A p-TAK S412

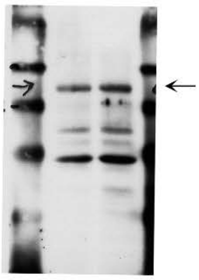

Fig. 5A p-TAK S184,187

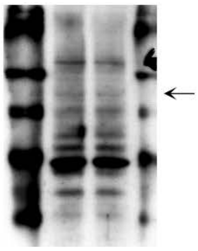

Fig. 5A TAK

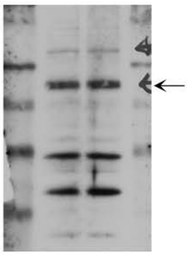

Fig. 5A p-JNK

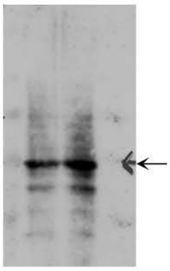

Fig. 5A JNK

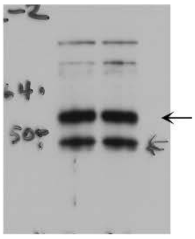

Fig. 5A p-p38

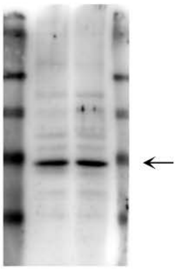

Fig. 5A p38

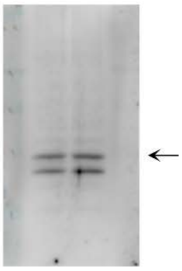

Fig. 5A p-Smad3 S423,425

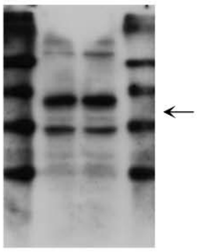

Fig. 5A p-Smad3 S208

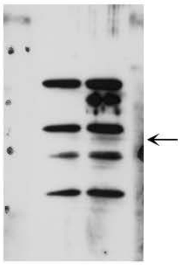

Fig. 5A Smad3

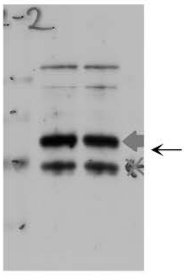

Fig. 5A a-SMA

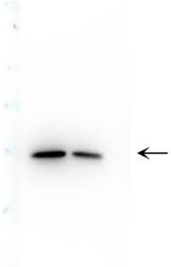

Fig. 5A tubulin

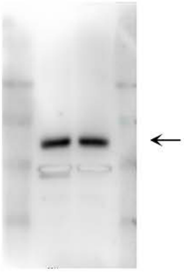

Fig. 5B p-Smad3 S208

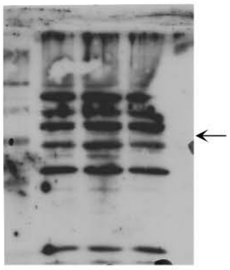

Fig. 5B Smad3

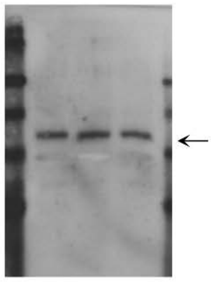

Fig. 5B p-JNK

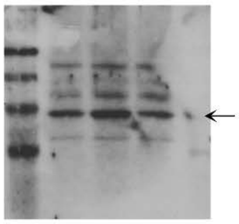

Fig. 5B JNK

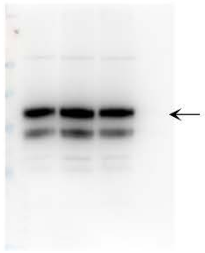

Fig. 5B a-SMA

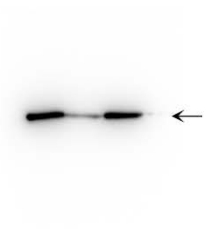

Fig. 5B tubulin

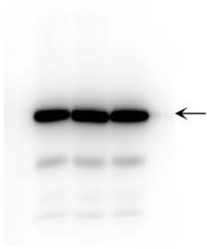

Fig. 5C p-Smad3 S208

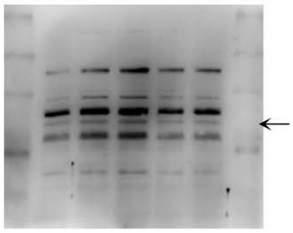

Fig. 5C tubulin

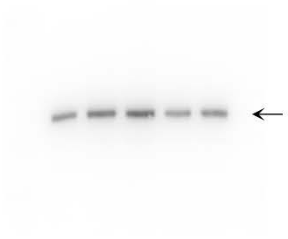

Fig. 6B nuclear Smad3

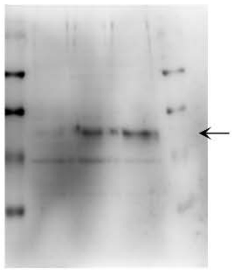

Fig. 6B nuclear lamin A/C

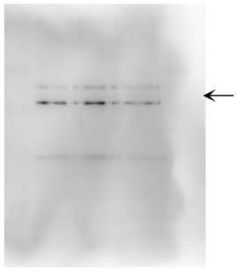

Fig. 6C cytoplasmic Smad3

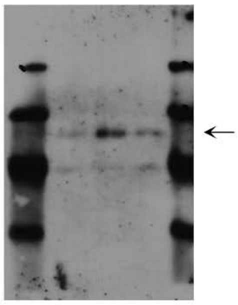

Fig. 6C cytoplasmic tubulin

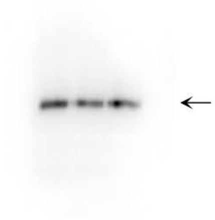

Fig. S2

lane1. HSCs-P1
lane2. albumin(1h)
lane3. albumin(4h)
lane4. HSCs-P1

Fig. S2 p-TAK1(S412)

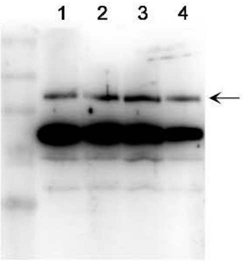

Fig. S2 TAK1

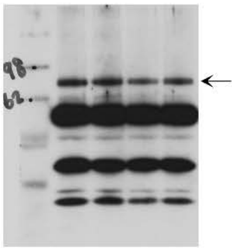

Fig. S2 p-JNK

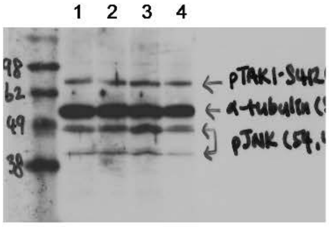

Fig. S2 JNK

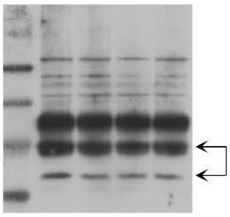

Fig. S2 p-Smad3(S208)

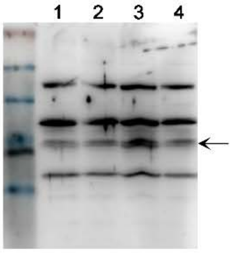

Fig. S2 Smad3

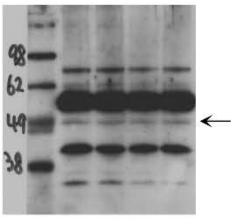

Fig. S2 tubulin

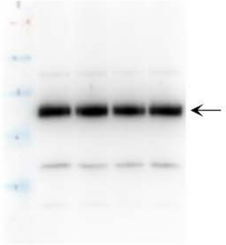

Supplementary Table S1. Primers used in this study

Gene	Primer	Sequence
ACTA2 (α-SMA)	Forward	5'- CCAGCACCATGAAGATCAAG -3'
	Reverse	5'- TGGAAGGTAGACAGCGAAGC -3'
Col1a1 (collagen 1)	Forward	5'- CGACCTCAAGATGTGCCACT -3'
	Reverse	5'- CTTGGTTAGGGTCGATCCAG -3'
IL-α	Forward	5'- CACTTGGTTAAATGACCTGCAA -3'
	Reverse	3'- AACACGGGCTGGTCTTCTC -3'
IL-1β	Forward	5'- GAAATGCCACCTTTTGACAGTG -3'
	Reverse	3'- TGGATGCTCTCATCAGGACAG -3'
IL-6	Forward	5'- AACGATGATGCACTTGCAGA -3'
	Revese	3'- CTCTGAAGGACTCTGGCTTTG -3'
TNF-α	Forward	5'- CAAATGGCCTCCCTCTCAT -3'
	Reverse	3'- AGCTGCTCCTCCACTTGGT -3'
Smad7	Forward	5'- CAGGCTGTCCAGATGCTGT -3'
	Reverse	5'- CCAGGCTCCAGAAGAAGTTG -3'
TGF-β1 R2	Forward	5'- GGGCGAGACTTTCTTCATGT -3'
	Reverse	5'- TGACACCCGTCACTTGGATA -3'
Importin7	Forward	5'- ATGGATCCCAACACCATCAT -3'
	Reverse	5'- TCTGGAGCAGTGTTGAAACG -3'
Importin8	Forward	5'- GCCATGGGGATTTTACACAC -3'
	Reverse	5'- TCAATGATTCGTAGGCAGATG -3'
GAPDH	Forward	5'- TCAACAGCAACTCCCACTCTTCCA -3'
	Reverse	5'- TTGTCATTGAGAGCAATGCCAGCC -3'
